# The effect of mitochondrial calcium uniporter and cyclophilin D knockout on resistance of brain mitochondria to Ca^2+^-induced damage

**DOI:** 10.1016/j.jbc.2021.100669

**Published:** 2021-04-16

**Authors:** James Hamilton, Tatiana Brustovetsky, Nickolay Brustovetsky

**Affiliations:** 1 Department of Pharmacology and Toxicology, Indiana University School of Medicine, Indianapolis, Indiana, USA; 2 Stark Neurosciences Research Institute, Indiana University School of Medicine, Indianapolis, Indiana, USA

**Keywords:** mitochondria, respiration, calcium, membrane potential, permeability transition pore, mitochondrial calcium uniporter, cyclophilin D, BSA, bovine serum albumin, CsA, cyclosporin A, CyD, cyclophilin D, EGTA, ethylene glycol tetraacetic acid, FFA, free fatty acid, IMM, inner mitochondrial membrane, MCU, mitochondrial calcium uniporter, MCU/CyD-DKO, MCU/CyD-double KO, PLA2, phospholipase A2, PTP, permeability transition pore, TPP^+^, tetraphenylphosphonium

## Abstract

The mitochondrial calcium uniporter (MCU) and cyclophilin D (CyD) are key players in induction of the permeability transition pore (PTP), which leads to mitochondrial depolarization and swelling, the major signs of Ca^2+^-induced mitochondrial damage. Mitochondrial depolarization inhibits ATP production, whereas swelling results in the release of mitochondrial pro-apoptotic proteins. The extent to which simultaneous deletion of MCU and CyD inhibits PTP induction and prevents damage of brain mitochondria is not clear. Here, we investigated the effects of MCU and CyD deletion on the propensity for PTP induction using mitochondria isolated from the brains of MCU-KO, CyD-KO, and newly created MCU/CyD-double knockout (DKO) mice. Neither deletion of MCU nor of CyD affected respiration or membrane potential in mitochondria isolated from the brains of these mice. Mitochondria from MCU-KO and MCU/CyD-DKO mice displayed reduced Ca^2+^ uptake and diminished extent of PTP induction. The Ca^2+^ uptake by mitochondria from CyD-KO mice was increased compared with mitochondria from WT mice. Deletion of CyD prevented mitochondrial swelling and resulted in transient depolarization in response to Ca^2+^, but it did not prevent Ca^2+^-induced delayed mitochondrial depolarization. Mitochondria from MCU/CyD-DKO mice did not swell in response to Ca^2+^, but they did exhibit mild sustained depolarization. Dibucaine, an inhibitor of the Ca^2+^-activated mitochondrial phospholipase A2, attenuated and bovine serum albumin completely eliminated the sustained depolarization. This suggests the involvement of phospholipase A2 and free fatty acids. Thus, in addition to induction of the classical PTP, alternative deleterious mechanisms may contribute to mitochondrial damage following exposure to elevated Ca^2+^.

Mitochondrial Ca^2+^ uptake plays an important role in the maintenance of cellular functions and in the regulation of mitochondrial activities (1, 2). Calcium accumulation by mitochondria is involved in maintaining low cytosolic Ca^2+^ and in stimulating mitochondrial respiration by activating mitochondrial dehydrogenases such as pyruvate, α-ketoglutarate, and isocitrate dehydrogenases and, thus stimulates mitochondrial respiration and oxidative phosphorylation (3). On the other hand, excessive Ca^2+^ accumulation by mitochondria may lead to induction of the mitochondrial permeability transition pore (PTP), the major mechanism of Ca^2+^-induced mitochondrial damage (4, 5).

Ca^2+^ uptake by mitochondria is mediated by the mitochondrial Ca^2+^ uniporter complex which consists of several components including MCU (mitochondrial calcium uniporter) (6, 7), MCUb (8), MICU1 and MICU2 (9, 10, 11), EMRE (12), and MCUR (13). The MCU is considered to be the Ca^2+^ channel in the inner mitochondrial membrane (IMM) (7). Deletion of MCU fully inhibited Ca^2+^ uptake by liver, skeletal muscle, and heart mitochondria, thus precluding PTP induction (14). On the other hand, MCU deletion in brain mitochondria inhibited but failed to completely prevent Ca^2+^ uptake and, consequently, did not completely prevent PTP induction (14, 15).

PTP induction caused by excessive Ca^2+^ accumulation is the major mechanism of Ca^2+^-induced mitochondrial damage (5). Induction of the PTP leads to mitochondrial depolarization and swelling of the organelles (16). In addition to PTP, mitochondrial depolarization can be induced by free fatty acids (FFAs) (17, 18, 19). Deletion of MCU in brain mitochondria diminished but did not completely prevent mitochondrial swelling and depolarization (14). In our previous study with brain mitochondria from MCU-KO mice, we showed that a combination of PTP inhibitors, cyclosporin A (CsA), ADP, and bovine serum albumin (BSA, free from FFA), completely inhibited mitochondrial swelling induced by Ca^2+^, thereby attributing mitochondrial swelling to PTP induction (14). However, in this previous study, we did not investigate the effect of PTP inhibitors on Ca^2+^-induced mitochondrial depolarization (14).

The molecular composition of the PTP is still an area of active research and zealous debate (20). However, there is a consensus regarding the involvement of mitochondrial cyclophilin D (CyD) in the PTP (21, 22, 23, 24). It has also been postulated that CyD sensitizes PTP to Ca^2+^ (24). Early studies established that CyD is a molecular target for CsA, and application of CsA to isolated mitochondria increases mitochondrial Ca^2+^ uptake and defers PTP induction (25, 26). Moreover, deletion of CyD increases resistance of mitochondria to Ca^2+^-induced damage and, correspondingly, augments Ca^2+^ retention capacity and protects mitochondrial membrane potential (21, 22, 23, 24).

In the present study, we compared the functions of brain mitochondria from CyD-KO and MCU-KO mice and their genetic backgrounds C57BL/6 and CD1 mice, respectively. In addition, we analyzed brain mitochondria from MCU/CyD-double KO (MCU/CyD-DKO) mice. We investigated, in particular, the effect of CyD deletion on PTP induction in brain mitochondria lacking MCU. Deletion of CyD prevented Ca^2+^-induced swelling of MCU-KO brain mitochondria but could not completely preclude Ca^2+^-induced sustained mitochondrial depolarization. The latter was attenuated by dibucaine, a phospholipase A2 inhibitor, and prevented by BSA, suggesting involvement of a phospholipase A2- and FFA-dependent mechanism of depolarization in Ca^2+^-treated brain mitochondria from MCU/CyD-DKO mice.

## Results

### Breeding and identification of MCU/CyD-DKO mice

**Figure 1:**
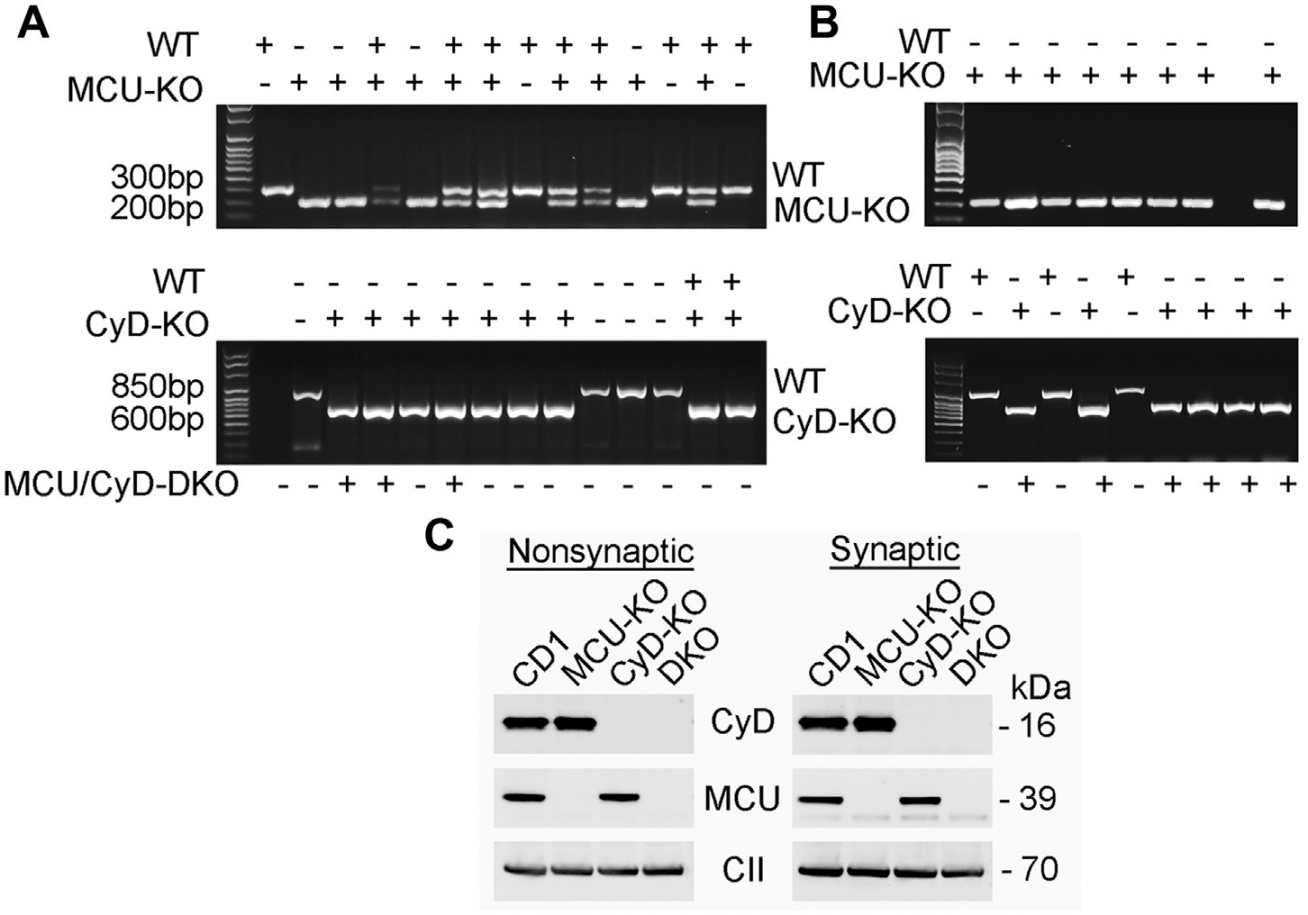
**Representative genotyping of CD1, MCU-KO, CyD-KO, and MCU/CyD-DKO mice and immunoblotting with brain mitochondria isolated from these mouse strains.** MCU/CyD-DKO mice used in our experiments were generated by crossing animals that were heterozygous for both MCU and CyD. *A*, representative genotyping data produced by PCR of DNA from tail tissue was used to identify mice that are MCU (*upper gel*) and CyD (*lower gel*) null. Samples from the same mice were used to determine the presence or absence of DNA encoding MCU and CyD. These samples were acquired from offspring obtained after crossing heterozygous MCU-KO and CyD-KO mice. *B*, representative genotyping data produced by PCR of DNA from tail tissue. Samples from the same mice were used to determine the presence or absence of DNA encoding MCU and CyD. These samples were acquired from F1 offspring obtained after crossing homozygous MCU-KO and CyD-KO mice. *C*, Western blotting of isolated brain nonsynaptic and synaptic mitochondria from CD1, MCU-KO, CyD-KO and MCU/CyD-DKO mice demonstrating the lack of MCU and CyD in brain mitochondria from DKO animals. Complex II, 70-kDa subunit (CII) was used as a loading control. CyD, cyclophilin D; MCU, mitochondrial calcium uniporter; MCU/CyD-DKO, MCU/CyD-double KO.

In our experiments, we used MCU-KO and CyD-KO mice, and their genetic backgrounds CD1 and C57BL/6 mice, respectively. In addition, we generated MCU/CyD-DKO mice by crossing MCU-KO mice with CyD-KO mice and then interbreeding the resultant F1 progeny to produce mice that were null for both MCU and CyD. Every mouse used in our experiments was genotyped. Figure 1, *A* and *B* show representative genotyping data of tail tissue from WT, MCU-KO, CyD-KO, and MCU/CyD-DKO mice. Samples from the same mice were used to determine the presence or absence of DNA encoding MCU (upper panel) and CyD (lower panel). In addition, immunoblotting was used to confirm the absence of MCU and CyD in all mitochondrial preparations used in our experiments (Fig. 1*C*).

### Ca^2+^ retention capacity of brain nonsynaptic and synaptic mitochondria

**Figure 2:**
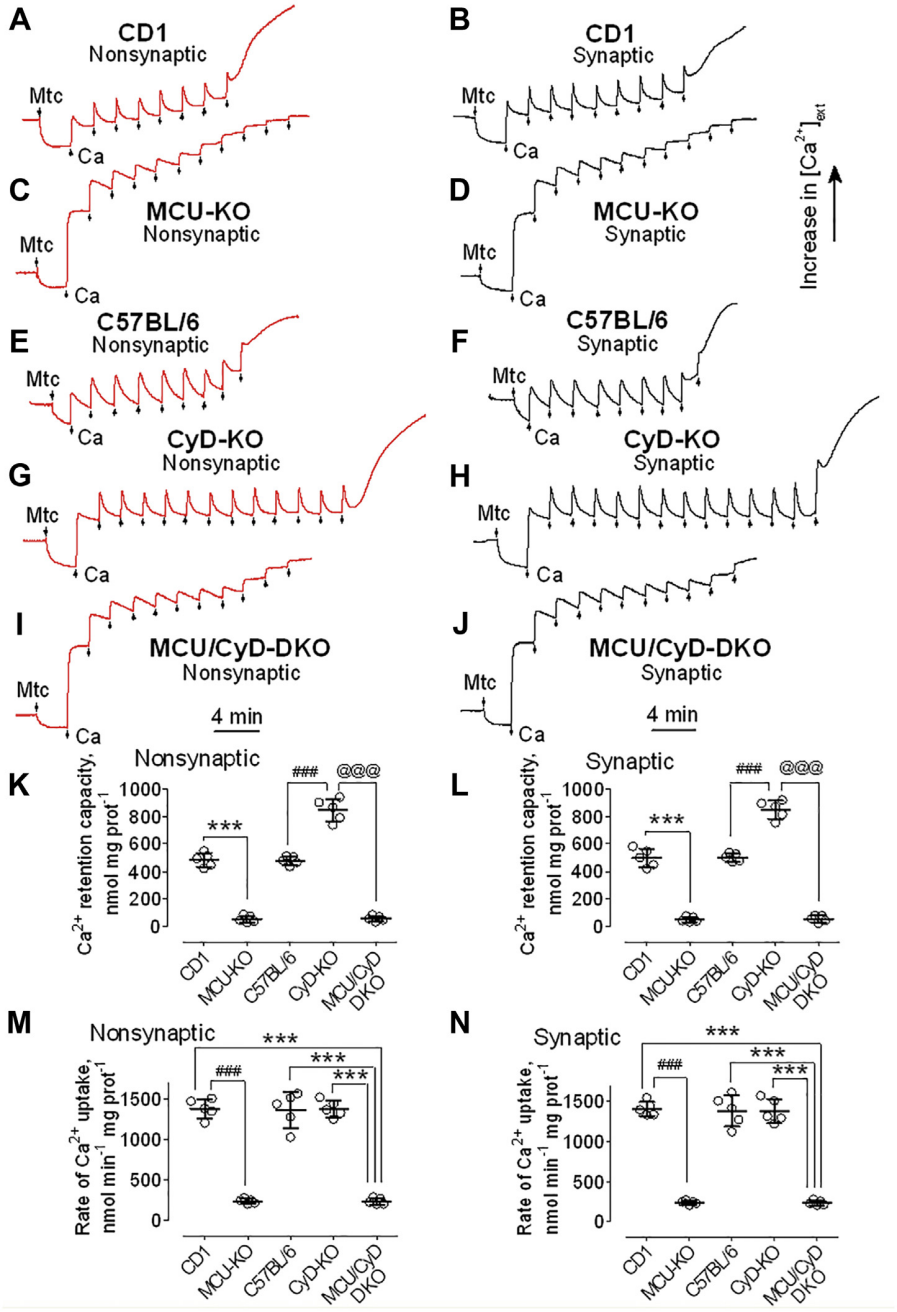
**Ca**^**2+**^**retention capacities and rates of Ca**^**2+**^**uptake by brain nonsynaptic and synaptic mitochondria isolated from CD1, MCU-KO, C57BL/6, CyD-KO, and MCU/CyD-DKO mice.** Ca^2+^ uptake was assessed in brain nonsynaptic (*A*, *C*, *E*, *G*, *I*, *red traces*) and synaptic (*B*, *D*, *F*, *H*, *J*, *black traces*) mitochondria incubated at 37 °C in the standard incubation medium supplemented with the Complex I substrates pyruvate (3 mM) plus malate (1 mM). Where indicated nonsynaptic or synaptic mitochondria (Mtc), both 60 μg of protein, were added. Where indicated, 10 μM Ca^2+^ (delivered as CaCl_2_) was applied to brain mitochondria from CD1 (*A* and *B*), MCU-KO (*C* and *D*), C57BL/6 (*E* and *F*), CyD-KO (*G* and *H*), and MCU/CyD-DKO (*I* and *J*) mice. In all experiments, incubation medium was additionally supplemented with 100 μM ADP and 1 μM oligomycin (65). *K* and *L*, Ca^2+^ retention capacity data are summarized for experiments with nonsynaptic (*K*) and synaptic (*L*) mitochondria, respectively. Data are mean ± SD from five separate experiments. ∗∗∗*p* < 0.001 comparing Ca^2+^ retention capacity of MCU-KO and CD1 Mtc, for both nonsynaptic and synaptic Mtc (ANOVA *p* < 0.0001, F = 246.9 for nonsynaptic Mtc and F = 266.0 for synaptic Mtc); ^###^*p* < 0.001 comparing Ca^2+^ retention capacity of CyD-KO and C57BL/6 Mtc, for both nonsynaptic and synaptic Mtc (ANOVA *p* < 0.0001, F = 246.9 for nonsynaptic Mtc and F = 266.0 for synaptic Mtc); ^@@@^*p* < 0.001 comparing Ca^2+^ retention capacity of MCU/CyD-DKO and CyD-KO Mtc, for both nonsynaptic and synaptic Mtc (ANOVA *p* < 0.0001, F = 246.9 for nonsynaptic Mtc and F = 266.0 for synaptic Mtc). *M* and *N*, rates of Ca^2+^ uptake by brain nonsynaptic and synaptic mitochondria, respectively, isolated from CD1, MCU-KO, C57BL/6, CyD-KO, and MCU/CyD-DKO mice. Ca^2+^ uptake rates were quantified from experiments shown in Figure 2 as Ca^2+^ uptake per minute per mg of mitochondrial protein. Data are mean ± S.D. from five separate experiments. ^###^*p* < 0.001 comparing rates of Ca^2+^ uptake by mitochondria from CD1 and MCU-KO mice (ANOVA *p* < 0.0001, F = 128.9 for nonsynaptic Mtc and F = 143.6 for synaptic Mtc); ∗∗∗*p* < 0.001 comparing rates of Ca^2+^ uptake by mitochondria from CD1, C57BL/6, and CyD-KO mice and by mitochondria from MCU/CyD-DKO mice (ANOVA *p* < 0.0001, F = 128.9 for nonsynaptic Mtc and F = 143.6 for synaptic Mtc). CyD, cyclophilin D; MCU, mitochondrial calcium uniporter; MCU/CyD-DKO, MCU/CyD-double KO.

We used a Ca^2+^-sensitive electrode to evaluate Ca^2+^ uptake by isolated Percoll-purified brain mitochondria. Ca^2+^ retention capacity (also known as Ca^2+^ uptake capacity (27, 28, 29, 30)) was assessed by applying multiple pulses of 10 μM Ca^2+^ delivered as CaCl_2_ (Fig. 2). Mitochondrial Ca^2+^ uptake was monitored by following a decrease in Ca^2+^ concentration in the incubation medium outside of mitochondria. Despite complete deletion of MCU in MCU-KO and MCU/CyD-DKO mice (Fig. 1*C*), brain mitochondria from these animals were able to take Ca^2+^ up (Fig. 2, *C*, *D* and *I*–*L*). Similar results were recently reported for mitochondria isolated from photoreceptor cells (31). Consistent with our previously reported data (14), brain mitochondria isolated from CD1 mice accumulated Ca^2+^ at a six times higher rate than brain mitochondria isolated from MCU-KO mice (Fig. 2, *M* and *N*). The total amount of accumulated Ca^2+^ was nine times smaller in brain mitochondria from MCU-KO mice compared with mitochondria from WT CD1 mice (Fig. 2, *A*–*D*, *K* and *L*). In contrast to the difference between CD1 and MCU-KO mitochondria, brain mitochondria from CyD-KO mice accumulated two times more Ca^2+^ compared with brain mitochondria from C57BL/6 mice, the genetic background for CyD-KO mice (Fig. 2, *E*–*H*, *K* and *L*). These results with CyD-KO mitochondria are consistent with our previously reported data that demonstrated increased Ca^2+^ uptake in brain mitochondria isolated from CyD-KO mice compared with mitochondria from C57BL/6 mice (26). Ca^2+^ retention capacity of mitochondria from MCU/CyD-DKO mice was similar to the Ca^2+^ retention capacity of mitochondria from MCU-KO mice (Fig. 2, *C*, *D* and *I*–*L*). Similar to MCU-KO mitochondria, the rate of Ca^2+^ uptake by MCU/CyD-DKO brain mitochondria was six times slower than the rates of Ca^2+^ uptake by brain mitochondria from CD1, C57BL/6, or CyD-KO mice (Fig. 2, *M* and *N*).

### Respiration of brain nonsynaptic and synaptic mitochondria

**Figure 3:**
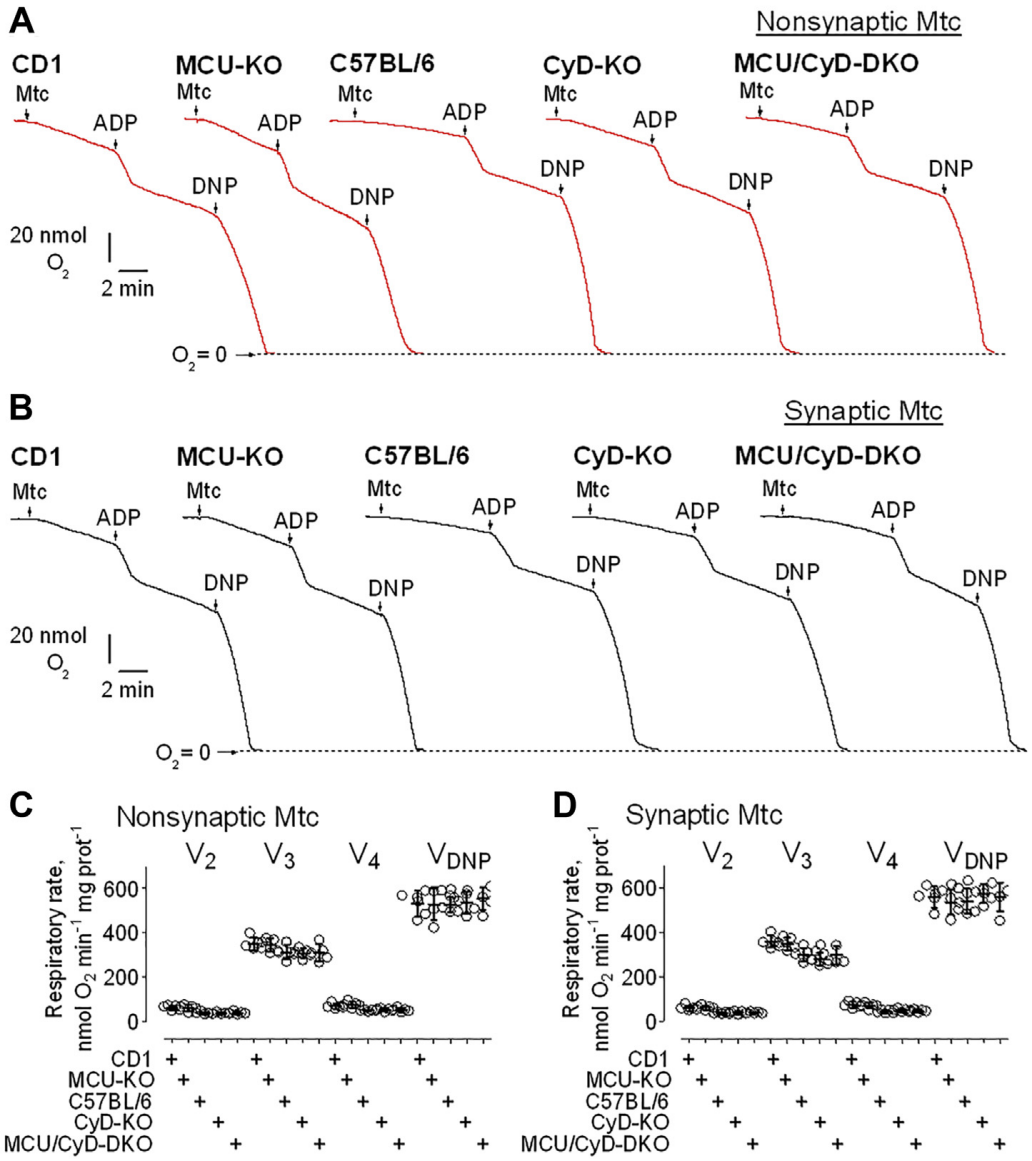
**Respiratory activities of brain nonsynaptic and synaptic mitochondria isolated from CD1, MCU-KO, C57BL/6, CyD-KO, and MCU/CyD-DKO mice.***A* and *B*, representative respiratory traces of mitochondrial O_2_ consumption for nonsynaptic (*A*, *red traces*) and synaptic (*B*, *black traces*) mitochondria, respectively, incubated at 37 °C in the standard incubation medium supplemented with pyruvate (3 mM) plus malate (1 mM) and 0.1% BSA (free from FFA). Where indicated nonsynaptic or synaptic mitochondria (Mtc), both 60 μg of protein, were added, followed by additions of 150 μM ADP and 60 μM 2,4-dinitrophenol (DNP). Averaged respiratory rates are summarized for brain nonsynaptic (*C*) and synaptic (*D*) mitochondria. Data are mean ± SD for five separate experiments. BSA, bovine serum albumin; CyD, cyclophilin D; FFA, free fatty acid; MCU, mitochondrial calcium uniporter; MCU/CyD-DKO, MCU/CyD-double KO.

**Figure 4:**
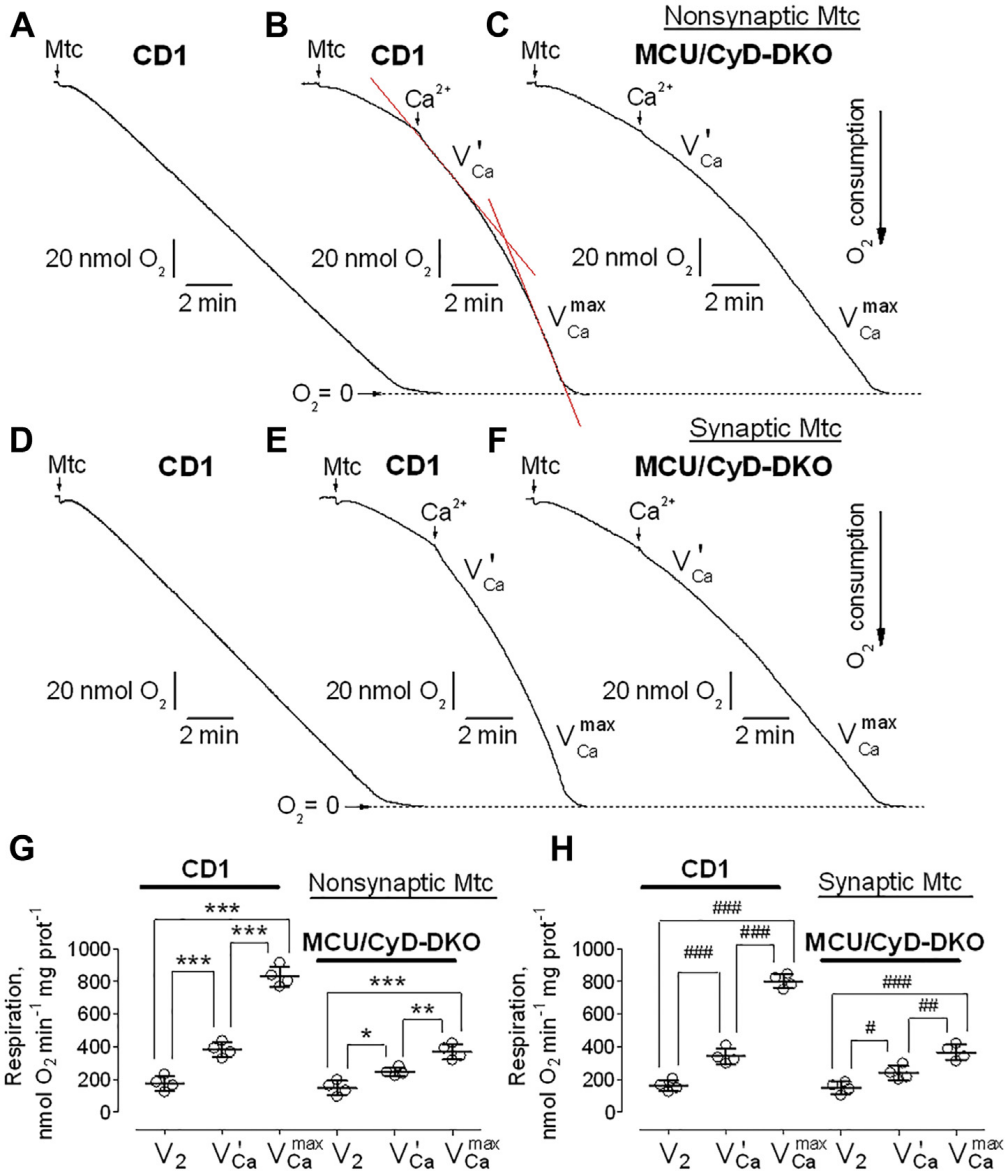
**Ca**^**2+**^**-stimulated respiration of brain nonsynaptic and synaptic mitochondria isolated from CD1 and MCU/CyD-DKO mice.***A*–*F*, representative respiratory traces of mitochondrial O_2_ consumption for nonsynaptic (*A*–*C*) and synaptic (*D*–*F*) mitochondria, respectively, incubated at 37 °C in the standard incubation medium supplemented with succinate (3 mM) plus glutamate (3 mM). Where indicated nonsynaptic or synaptic mitochondria (Mtc), both 60 μg of protein, were added, followed by additions of 100 μM CaCl_2_. *A* and *D*, the representative respiratory traces obtained with nonsynaptic (*A*) and synaptic (*D*) mitochondria isolated from brains of CD1 mice are shown. These experiments demonstrate linearity of oxygen electrode used in our study. *B*, *C*, *E*, and *F*, the respiratory rates were calculated using linear fragments of the respiratory traces (*red lines*) immediately after Ca^2+^ addition $\left({\text{V}}_{\text{Ca}}^{\text{′}}\right)$ and at the maximal O_2_ consumption $\left({\text{V}}_{\text{Ca}}^{\text{max}}\right)$. Averaged respiratory rates are summarized for brain nonsynaptic (*G*) and synaptic (*H*) mitochondria. Data are mean ± SD from four separate experiments. ∗∗∗*p* < 0.001 comparing respiratory rates of nonsynaptic mitochondria from CD1 mice (ANOVA *p* < 0.0001, F = 117.7); ∗∗*p* < 0.01 comparing ${\text{V}}_{\text{Ca}}^{\text{′}}$ and ${\text{V}}_{\text{Ca}}^{\text{max}}$ respiratory rates of nonsynaptic mitochondria from MCU/CyD-DKO mice (ANOVA *p* < 0.0001, F = 117.7); ∗*p* < 0.05 comparing V_2_ and ${\text{V}}_{\text{Ca}}^{\text{′}}$ respiratory rates of nonsynaptic mitochondria from MCU/CyD-DKO mice (ANOVA *p* < 0.0001, F = 117.7). ^###^*p* < 0.001 comparing respiratory rates of synaptic mitochondria from CD1 mice (ANOVA *p* < 0.0001, F = 129.4); ^##^*p* < 0.01 comparing ${\text{V}}_{\text{Ca}}^{\text{′}}$ and ${\text{V}}_{\text{Ca}}^{\text{max}}$ respiratory rates of synaptic mitochondria from MCU/CyD-DKO mice (ANOVA *p* < 0.0001, F = 129.4); ^#^*p* < 0.05 comparing V_2_ and ${\text{V}}_{\text{Ca}}^{\text{′}}$ respiratory rates of synaptic mitochondria from MCU/CyD-DKO mice (ANOVA *p* < 0.0001, F = 129.4). CyD, cyclophilin D; MCU, mitochondrial calcium uniporter; MCU/CyD-DKO, MCU/CyD-double KO.

The influx of Ca^2+^ into the mitochondrial matrix can activate pyruvate dehydrogenase and the TCA cycle enzymes isocitrate dehydrogenase and α-ketoglutarate dehydrogenase, thus stimulating mitochondrial respiration (3). Therefore, inhibition of Ca^2+^ uptake could affect mitochondrial respiration. To investigate the effect of suppressed Ca^2+^ uptake on mitochondrial respiration, we used a Clark-type oxygen electrode to measure the rate of oxygen consumption by mitochondria. We assessed basal respiration with only substrates present in the incubation medium (V_2_), respiration stimulated by ADP (V_3_), respiration after depletion of exogenous ADP (V_4_), and maximal, uncoupled respiration in the presence of the uncoupler 2,4-dinitrophenol (V_DNP_). In our experiments, we evaluated the respiratory rates of both nonsynaptic and synaptic mitochondria from CD1, C57BL/6, MCU-KO, CyD-KO, and MCU/CyD-DKO. We did not find a significant difference in mitochondrial respiration among brain mitochondria from any of these five mouse strains (Fig. 3, *A* and *B*). Statistical summary of respiratory data is shown in Figure 3, *C* and *D*. In subsequent experiments, we assessed the stimulating effect of Ca^2+^ on respiration of nonsynaptic and synaptic brain mitochondria from control, CD1 mice, and MCU/CyD-DKO mice (Fig. 4). Previously, we showed that Ca^2+^ could inhibit respiration of mitochondria oxidizing pyruvate plus malate but not succinate plus glutamate (14, 32). Consequently, in our present experiments, mitochondria were fueled with 3 mM succinate plus 3 mM glutamate. Predictably, the Ca^2+^-stimulated respiration of mitochondria from CD1 mice was more robust compared with Ca^2+^-stimulated respiration of mitochondria from MCU/CyD-DKO mice. Although basal respiration (V_2_) was not different in mitochondria from MCU/CyD-DKO *versus* mitochondria from CD1 mice, Ca^2+^-stimulated respiration assessed immediately after Ca^2+^ addition $\left({\text{V}}_{\text{Ca}}^{\text{′}}\right)$, and at the maximal O_2_ consumption, $\left({\text{V}}_{\text{Ca}}^{\text{max}}\right)$ was ∼50% slower, signifying the persistence of Ca^2+^ uptake at a slower rate in the DKO mitochondria.

### Ca^2+^ effects on membrane potential and mitochondrial swelling in brain mitochondria from CD1 and MCU-KO mice

**Figure 5:**
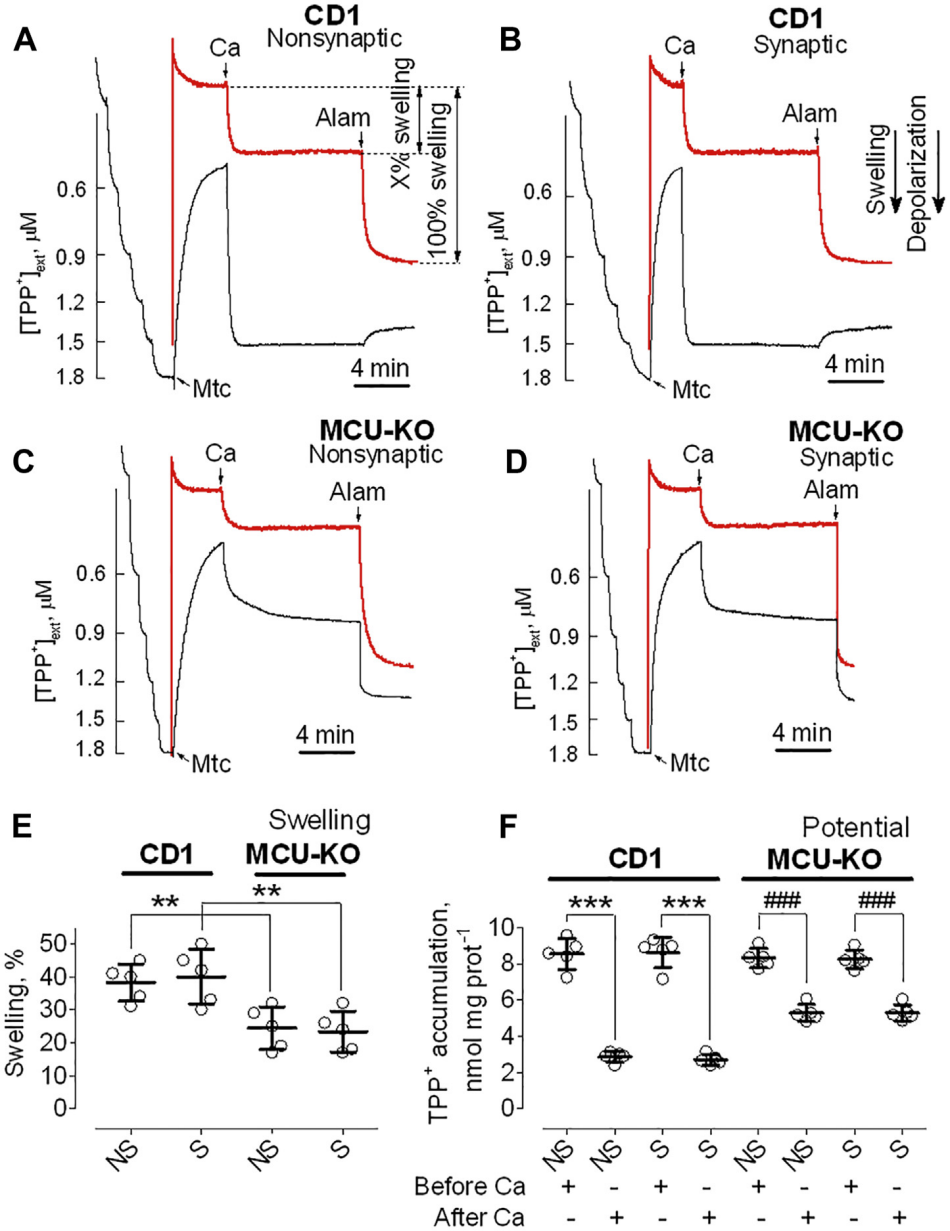
**Ca**^**2+**^**-induced depolarizations and mitochondrial swelling in brain mitochondria from CD1 and MCU-KO mice.** TPP^+^ concentration in the incubation medium (*black traces*), indicative of mitochondrial membrane potential, and light scattering (*red traces*), representative of mitochondrial swelling, were monitored in brain nonsynaptic (*A* and *C*) and synaptic (*B* and *D*) mitochondria incubated at 37 °C in the standard incubation medium supplemented with pyruvate (3 mM) plus malate (1 mM). Where indicated in representative TPP^+^ and light scattering traces, Ca^2+^ (100 μM) and alamethicin (30 μg/ml, Alam) were applied to brain mitochondria (Mtc) from CD1 (*A* and *B*) and MCU-KO (*C* and *D*) mice. The 100 μM Ca^2+^ pulse triggered PTP induction, and alamethicin induced maximal swelling. *E*, swelling data are summarized for experiments with nonsynaptic (NS) and synaptic (S) mitochondria. ∗∗*p* < 0.01 comparing swelling of synaptic and nonsynaptic Mtc from CD1 mice with synaptic and nonsynaptic Mtc from MCU-KO mice, respectively (ANOVA *p* < 0.0012, F = 8.645). *F*, TPP^+^ accumulation data are summarized before and after the addition of Ca^2+^ to brain mitochondria. Data are mean ± SD for five separate experiments. ∗∗∗*p* < 0.001 comparing TPP^+^ accumulation by CD1 Mtc, both synaptic and nonsynaptic, before and 10 min after Ca^2+^ addition (ANOVA *p* < 0.0001, F = 98.52); ^###^*p* < 0.001 comparing TPP^+^ accumulation by MCU-KO Mtc, both synaptic and nonsynaptic, before and 10 min after Ca^2+^ (ANOVA *p* < 0.0001, F = 98.52). CyD, cyclophilin D; MCU, mitochondrial calcium uniporter; MCU/CyD-DKO, MCU/CyD-double KO; PTP, permeability transition pore; TPP^+^, tetraphenylphosphonium.

Consistent with the ability of MCU-KO brain mitochondria to accumulate Ca^2+^ (Fig. 2, *C* and *D*), these mitochondria responded to a large Ca^2+^ pulse by swelling and membrane depolarization although to a lesser extent than mitochondria from CD1 mice (Fig. 5). In these and other similar experiments, we simultaneously monitored changes in mitochondrial swelling by following light scattering of the mitochondrial suspension as well as changes in mitochondrial membrane potential by measuring the distribution of the lipophilic cation tetraphenylphosphonium (TPP^+^) across the IMM. Addition of CaCl_2_ (100 μM) to brain mitochondria from CD1 and MCU-KO mice (Fig. 5) produced a decrease in light scattering, indicating mitochondrial swelling, and an increase in TPP^+^ concentration in the incubation medium ([TPP^+^]_ext_), indicating mitochondrial depolarization. The amount of mitochondrial swelling induced by Ca^2+^ was quantified as a percentage of maximal mitochondrial swelling induced by the pore-forming peptide alamethicin (33, 34). Addition of Ca^2+^ (100 μM) to brain mitochondria from MCU-KO mice produced approximately two times less swelling and depolarization compared with CD1 mitochondria (Fig. 5). The decreased amplitude of Ca^2+^-induced mitochondrial swelling and diminished depolarization suggested that mitochondria from MCU-KO mice have reduced propensity for PTP induction but are still capable of undergoing the permeability transition. Statistical summaries of mitochondrial swelling and membrane potential are shown in Figure 5, *E* and *F*, respectively.

### Ca^2+^ effects on membrane potential and mitochondrial swelling in brain mitochondria from C57BL/6 and CyD-KO mice

**Figure 6:**
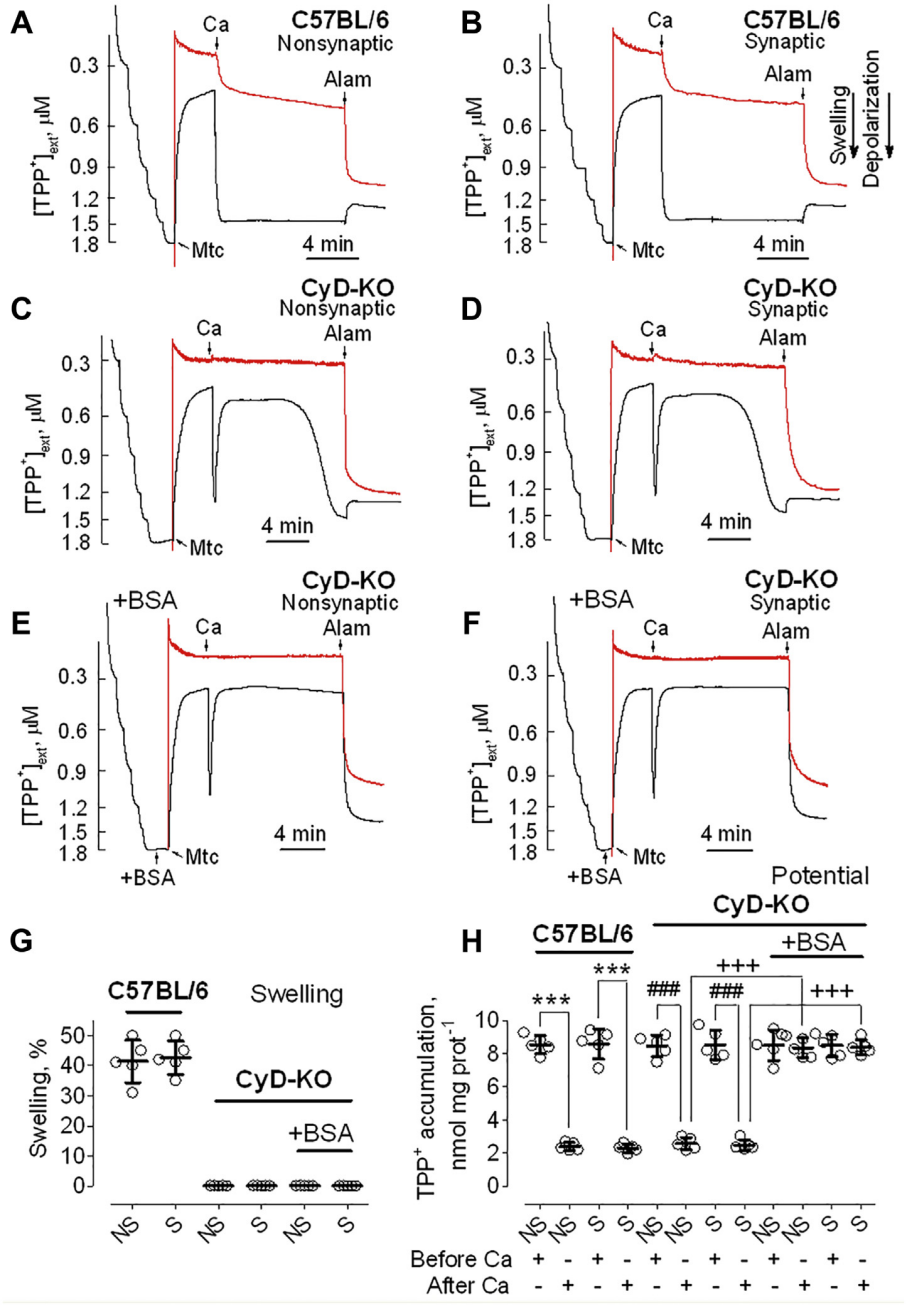
**Ca**^**2+**^**-induced depolarizations and mitochondrial swelling in brain mitochondria from C57BL/6 and CyD-KO mice.** BSA protects against Ca^2+^-induced delayed depolarization. TPP^+^ concentration in the incubation medium (*black traces*), indicative of mitochondrial membrane potential, and light scattering (*red traces*), representative of mitochondrial swelling, were monitored in brain nonsynaptic (*A*, *C* and *E*) and synaptic (*B*, *D* and *F*) mitochondria incubated at 37 °C in the standard incubation medium supplemented with pyruvate (3 mM) plus malate (1 mM). Where indicated in representative TPP^+^ and light scattering traces, Ca^2+^ (100 μM) and alamethicin (30 μg/ml, Alam) were applied to brain mitochondria (Mtc) from C57BL/6 (*A* and *B*) and CyD-KO mice (*C*–*F*). The 100 μM Ca^2+^ pulse was intended to trigger PTP induction and alamethicin induced maximal swelling. *E* and *F*, the incubation medium was additionally supplemented with 0.1% BSA which binds FFA (38). *G*, swelling data are summarized for experiments with nonsynaptic (NS) and synaptic (S) mitochondria. *H*, TPP^+^ accumulation data are summarized before and after the addition of Ca^2+^ to brain mitochondria. Data are mean ± SD for five separate experiments. ∗∗∗*p* < 0.001 comparing TPP^+^ accumulation in C57BL/6 Mtc, both synaptic and nonsynaptic, before and 12 min after Ca^2+^ addition (ANOVA *p* < 0.0001, F = 115.0); ^###^*p* < 0.001 comparing TPP^+^ accumulation in CyD-KO Mtc, both synaptic and nonsynaptic, before and 12 min after Ca^2+^ addition (ANOVA *p* < 0.0001, F = 115.0); ^+++^*p* < 0.001 comparing TPP^+^ accumulation in CyD-KO Mtc, both synaptic and nonsynaptic, 12 min after Ca^2+^ addition, incubated with and without BSA (ANOVA *p* < 0.0001, F = 115.0). BSA, bovine serum albumin; CyD, cyclophilin D; PTP, permeability transition pore; TPP^+^, tetraphenylphosphonium.

Application of 100 μM Ca^2+^ to nonsynaptic (Fig. 6*A*) and synaptic (Fig. 6*B*) mitochondria isolated from C57BL/6 mice resulted in swelling of the organelles and caused a rapid, sustained depolarization. On the other hand, when applied to nonsynaptic (Fig. 6*C*) and synaptic (Fig. 6*D*) mitochondria from CyD-KO mice, a 100 μM Ca^2+^ pulse did not result in mitochondrial swelling but did cause a rapid, transient depolarization. Within 5 min following addition of Ca^2+^, CyD-KO mitochondria became repolarized to a level approaching the membrane potential before Ca^2+^ addition. However, later, without any additional Ca^2+^ application, CyD-KO mitochondria became gradually depolarized, without any measurable corresponding change in swelling and were completely depolarized 12 min after Ca^2+^ application (Fig. 6, *C* and *D*). Predictably, this delayed depolarization was not sensitive to CsA (not shown) and, therefore, could not be attributed to induction of the classical PTP. Another possibility was the uncoupling action of FFA. We hypothesized that Ca^2+^ accumulated in mitochondria could activate mitochondrial Ca^2+^-dependent phospholipase A2 (PLA2), which produces FFA (35, 36) that depolarize mitochondria (37). BSA binds FFA (38) and eliminates the effects of FFA on mitochondria (39). Thus, the use of BSA is the most effective way of removing FFA from mitochondria and protecting the organelles from deleterious effects of FFA.

Indeed, addition of BSA completely prevented the Ca^2+^-induced delayed mitochondrial depolarization of CyD-KO mitochondria (Fig. 6, *E* and *F*). Statistical summaries of measurements of mitochondrial swelling and membrane potential are shown in Figure 6, *G* and *H*, respectively.

### Effect of CyD deletion on PTP induction in brain mitochondria lacking MCU

**Figure 7:**
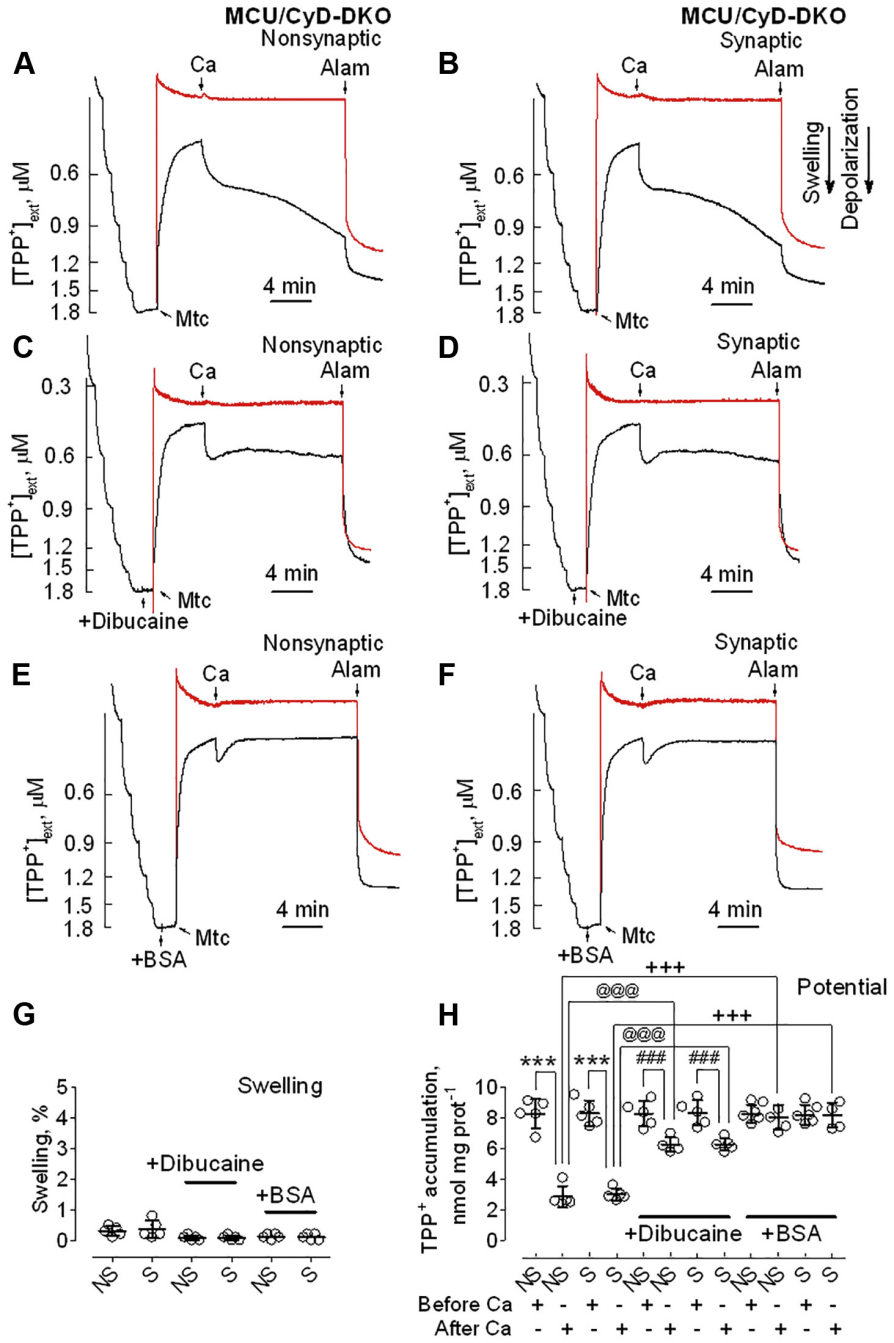
**Ca**^**2+**^**effects on membrane potential and mitochondrial swelling in brain mitochondria from MCU/CyD-DKO mice.** Dibucaine and BSA protect against Ca^2+^-induced sustained depolarization. TPP^+^ concentration in the incubation medium (*black traces*), indicative of mitochondrial membrane potential, and light scattering (*red traces*), representative of mitochondrial swelling, were monitored in MCU/CyD-DKO mouse-derived brain nonsynaptic (*A*, *C* and *E*) and synaptic (*B*, *D* and *F*) mitochondria incubated at 37 °C in the standard incubation medium supplemented with pyruvate (3 mM) plus malate (1 mM). *C* and *D*, where indicated, 50 μM dibucaine, an inhibitor of mitochondrial PLA2 (40), was added to brain mitochondria (Mtc) from MCU/CyD-DKO mice. *E* and *F*, the incubation medium was supplemented with 0.1% BSA, which binds FFA (38). Where indicated, Ca^2+^ (100 μM) and alamethicin (30 μg/ml, Alam) were applied to brain mitochondria (Mtc) from MCU/CyD-DKO mice (*A*–*F*). The 100 μM Ca^2+^ pulse was intended to trigger PTP induction and alamethicin induced maximal swelling. *G*, swelling data are summarized for experiments with nonsynaptic (NS) and synaptic (S) mitochondria. *H*, TPP^+^ accumulation data are summarized before and after the addition of Ca^2+^ to brain mitochondria. *G* and *H*, 50 μM dibucaine or 0.1% BSA (free from fatty acids) were present in the incubation medium. Data are mean ± SD for five separate experiments. ∗∗∗*p* < 0.001 comparing TPP^+^ accumulation in MCU/CyD-DKO Mtc, both synaptic and nonsynaptic, before and 12 min after Ca^2+^ addition (ANOVA *p* < 0.0001, F = 42.32); ^###^*p* < 0.001 comparing TPP^+^ accumulation in MCU/CyD-DKO Mtc, both synaptic and nonsynaptic, before and 12 min after Ca^2+^ addition, in the presence of dibucaine (ANOVA *p* < 0.0001, F = 42.32); ^@@@^*p* < 0.001 comparing TPP^+^ accumulation in MCU/CyD-DKO Mtc, both synaptic and nonsynaptic, 12 min after Ca^2+^ addition, incubated with and without dibucaine (ANOVA *p* < 0.0001, F = 42.32); ^+++^*p* < 0.001 comparing TPP^+^ accumulation in MCU/CyD-DKO Mtc, both synaptic and nonsynaptic, 12 min after Ca^2+^ addition, incubated with and without BSA (ANOVA *p* < 0.0001, F = 42.32). BSA, bovine serum albumin; CyD, cyclophilin D; MCU, mitochondrial calcium uniporter; MCU/CyD-DKO, MCU/CyD-double KO; PTP, permeability transition pore; TPP^+^, tetraphenylphosphonium.

It has been shown previously that CyD sensitizes mitochondria to induction of Ca^2+^-activated PTP and that genetic ablation of CyD deferred Ca^2+^-triggered permeability transition, resulting in increased Ca^2+^ uptake capacity in isolated mitochondria (24, 26). Therefore, we hypothesized that combined knockout of MCU and CyD may further decrease the already diminished propensity to PTP induction that was observed in brain mitochondria from MCU-KO mice (Fig. 5). Addition of 100 μM Ca^2+^ to MCU/CyD-DKO nonsynaptic (Fig. 7*A*) and synaptic (Fig. 7*B*) mitochondria produced no mitochondrial swelling, but did induce moderate sustained mitochondrial depolarization, from which membrane potential did not recover. The sustained depolarization of MCU/CyD-DKO mitochondria following Ca^2+^ application was, to some extent, surprising, and the mechanism was not completely clear. Similar to CyD-KO mitochondria (Fig. 6), the sustained depolarization was not sensitive to CsA (not shown) and, therefore, could not be attributed to classical PTP induction. Here again, we hypothesized that Ca^2+^ in mitochondria could activate PLA2, producing FFA that depolarize mitochondria (35, 36, 37). Indeed, dibucaine (50 μM), an inhibitor of mitochondrial PLA2 (40), significantly, but not completely, attenuated sustained depolarization caused by Ca^2+^, suggesting activation of PLA2 and its involvement in the Ca^2+^-induced depolarization (Fig. 7, *C*, *D* and *H*). Further support of this hypothesis came from experiments with BSA. Ca^2+^ application to MCU/CyD-DKO mitochondria incubated in the presence of BSA resulted in a transient Ca^2+^-induced depolarization followed by mitochondrial repolarization to pre-Ca^2+^ membrane potential (Fig. 7, *E* and *F*). Thus, BSA completely prevented sustained Ca^2+^-induced mitochondrial depolarization of MCU/CyD-DKO mitochondria. Statistical summaries for mitochondrial swelling and membrane potential evaluated in these experiments are shown in Figure 7, *G* and *H*, respectively. The protective effect of BSA suggests that the sustained Ca^2+^-induced depolarization observed in MCU/CyD-DKO brain mitochondria following Ca^2+^ application most likely was because of the uncoupling action of FFA.

## Discussion

In the present study, we investigated the effect of genetic ablation of the MCU and CyD on mitochondrial respiration, membrane potential, and induction of the PTP. Deletion of MCU or CyD alone or in combination did not affect mitochondrial respiration. The lack of changes in ADP-stimulated respiration (V_3_) suggests the absence of alterations in oxidative phosphorylation. The lack of changes in V_4_ indicates unaffected proton permeability of the mitochondrial inner membrane. This conclusion is supported by the lack of difference in membrane potential in mitochondria from all strains of mice used in our study. Finally, the lack of changes in DNP-stimulated respiration (V_DNP_) indicates unaffected maximal respiratory activity. Thus, these results suggest that MCU and CyD do not play an essential role in maintenance of respiratory activity in mitochondria isolated from brains of the tested mouse strains. On the other hand, Ca^2+^-induced stimulation of respiration of MCU/CyD-DKO mitochondria indicates that these mitochondria can take Ca^2+^ up, resulting in increased permeability of the mitochondrial inner membrane and subsequent mitochondrial depolarization.

Interesting peculiarities were noted in Ca^2+^ uptake experiments. With MCU-KO and MCU/CyD-DKO mitochondria, Ca^2+^ uptake during the first Ca^2+^ pulse seems stalled, but it resumes with the second Ca^2+^ pulse. We observed similar phenomenon in our previous study (14). It has to be noted that Ca^2+^ uptake during the first Ca^2+^ pulse seems deficient to various extents in mitochondria from all other mouse strains. The cause of this deficiency is not completely clear. This could reflect the need for reaching a certain Ca^2+^ threshold, from which Ca^2+^ uptake prevails over simultaneous Ca^2+^ efflux (41). In addition, MCU may require some Ca^2+^ to become active (11, 42, 43, 44). In our experiments, isolated mitochondria were stored on ice in the presence of 100 μM ethylene glycol tetraacetic acid (EGTA), and the incubation medium contained 10 μM EGTA. Consequently, mitochondria were stored and initially incubated in low Ca^2+^ medium. Therefore, MCU was probably initially inactive, and mitochondria were unable to actively take Ca^2+^ up during the first Ca^2+^ pulse but became more active in Ca^2+^ uptake during the second and subsequent Ca^2+^ pulses.

In our experiments, we found a drastic difference at the end of Ca^2+^ uptake experiments between mitochondria with MCU (CD1, C57BL/6, and CyD-KO) and mitochondria without MCU (MCU-KO and MCU/CyD-DKO). In the former, Ca^2+^ uptake is followed by the massive release of Ca^2+^ accumulated by mitochondria, whereas in the latter, mitochondria do not synchronously release accumulated Ca^2+^. The exact cause for this difference is not clear. It is possible that the lack of Ca^2+^ release could be because of the lack of PTP induction in mitochondria that are devoid of MCU. In this case, Ca^2+^ uptake in these mitochondria could be halted by a yet unknown mechanism. Alternatively, the lack of Ca^2+^ release could be explained by the lack of significant Ca^2+^ accumulation in MCU-KO mitochondria. The ability of mitochondria to accumulate Ca^2+^ depends on the rates of Ca^2+^ uptake and Ca^2+^ efflux from mitochondria (41). It is possible that the slow Ca^2+^ uptake by MCU-KO mitochondria could be compensated to some extent by simultaneous Ca^2+^ release. As a result, these mitochondria might be not capable of significant Ca^2+^ accumulation and, consequently, could not undergo massive Ca^2+^ release at the end of Ca^2+^ uptake experiments. Yet, it is conceivable that the PTP could be asynchronously induced in MCU-KO mitochondria, leading to a slow release of accumulated Ca^2+^ and gradual increase in external Ca^2+^, as seen in our experiments with MCU-deficient mitochondria.

The molecular identity of the MCU-independent Ca^2+^ uptake by brain mitochondria still is not clear. In our previous study, we tested some hypotheses regarding the molecular identity of the MCU-independent Ca^2+^ uptake machinery in brain mitochondria (14). We found that Ca^2+^ uptake in MCU-KO brain mitochondria was insensitive to CGP37157, an inhibitor of mitochondrial Na^+^/Ca^2+^ exchanger (45), and to dantrolene, an antagonist of ryanodine receptor, which may be present in mitochondria (46, 47). Yet, MCU-independent Ca^2+^ uptake by brain mitochondria could be completely inhibited by Ru360 (14). In the present study, we did not expand our search for the molecular identity of the MCU-independent Ca^2+^ uptake mechanism in brain mitochondria, and therefore, this issue still remains open.

Induction of the PTP is implicated in various pathologies (48). Ca^2+^ overload of mitochondria is the major mechanism leading to PTP induction (5, 20). Complete inhibition of mitochondrial Ca^2+^ uptake by deleting MCU is an effective way to prevent PTP induction in mitochondria from various noncerebral tissues. However, in brain mitochondria, deletion of MCU does not completely prevent Ca^2+^ uptake (14). Consequently, despite decreased Ca^2+^ uptake, brain mitochondria are still able to undergo PTP induction in response to Ca^2+^ application.

There are two major manifestations of PTP induction—mitochondrial swelling and depolarization (5). Deletion of MCU in brain mitochondria does not prevent Ca^2+^-induced mitochondrial swelling and depolarization, although it does decrease the extent of both (14). In the previous study, we showed that PTP inhibitors precluded mitochondrial swelling in MCU-KO brain mitochondria (14), attributing this swelling to PTP induction. However, in this previous study, we did not investigate the effect of PTP inhibitors on Ca^2+^-induced depolarization in MCU-KO brain mitochondria. Thus, the mechanism of the Ca^2+^-induced depolarization in MCU-KO brain mitochondria remained not completely understood.

Inhibition of the PTP by specific pharmacological inhibitors is an accepted method for attributing alterations in mitochondrial functions to PTP (5). CsA is the most prominent and widely used inhibitor of the PTP (49, 50). CsA binds to mitochondrial CyD and desensitizes mitochondria to harmful Ca^2+^ (51, 52). Alternatively, genetic ablation of mitochondrial CyD also desensitizes mitochondria to the deleterious effect of Ca^2+^ (24). The deletion of CyD significantly protected mitochondria from PTP induction and increased mitochondrial Ca^2+^ uptake capacity (21, 22, 23, 24). However, increasing mitochondrial Ca^2+^ load could overcome the protection conferred by CyD deletion (26). Consequently, we hypothesized that desensitizing mitochondria by genetic ablation of CyD and diminishing Ca^2+^ uptake because of MCU deletion might provide superior protection against Ca^2+^-induced mitochondrial damage. The results presented in this article demonstrate that our hypothesis was only partially correct.

Combined deletion of CyD and MCU in brain mitochondria completely prevented mitochondrial swelling but, in contrast to our expectations, only partially prevented mitochondrial depolarization. The residual mitochondrial depolarization was not sensitive to CsA, which was expected considering the complete deletion of CyD. On the other hand, BSA completely eliminated the sustained Ca^2+^-induced depolarization. Importantly, BSA also prevented delayed Ca^2+^-induced depolarization of CyD-KO brain mitochondria, suggesting that there might be some similarities between responses to Ca^2+^ by CyD-KO and MCU/CyD-DKO mitochondria.

BSA binds FFA (38), which can increase proton permeability of the inner mitochondrial membrane, leading to mitochondrial depolarization (37). Removal of FFA by BSA eliminates the deleterious effects of FFA on mitochondria (39). Mitochondria have PLA2 that can be activated by Ca^2+^ (53) and calcium-independent PLA2 (54, 55), which, in fact, can also be significantly activated by Ca^2+^ (56). Thus, it seems conceivable that stimulation of PLA2 by Ca^2+^ in CyD-KO as well as in MCU/CyD-DKO brain mitochondria could lead to increased production of FFA, leading to FFA-induced mitochondrial depolarization. Inhibition of sustained, Ca^2+^-induced mitochondrial depolarization by dibucaine, an inhibitor of PLA2 (40), supports this hypothesis. In addition, the protective effect of BSA distinctly links the sustained, Ca^2+^-induced depolarization to FFA. The mechanisms by which FFAs depolarize mitochondria following exposure to high Ca^2+^ are not completely understood. Such depolarization could be because of increased proton permeability of the IMM mediated by the interaction of FFA and adenine nucleotide translocase (57, 58) or because of the interaction of FFA with Ca^2+^, leading to formation of CsA-insensitive pores in the IMM (59).

Protection of mitochondria against Ca^2+^-induced damage with PTP inhibitors is considered a plausible therapeutic approach (48, 60) and, therefore, might help to alleviate cell injury and tissue damage in various pathologies. However, if focused solely on PTP inhibition, we may miss other potentially important deleterious mechanisms. Protection against PTP may result in increased accumulation of Ca^2+^ in mitochondria, subsequently leading to stimulation of mitochondrial PLA2 and an increased production of FFA (53). Consequently, FFA may depolarize mitochondria, leading to inhibition of oxidative phosphorylation and decline in ATP level (37). Our results strongly suggest this possibility and emphasize the need for a combinational approach in protecting mitochondria against Ca^2+^ overload and a comprehensive analysis of protective strategies that consider PTP-independent mechanisms of Ca^2+^-induced mitochondrial damage.

## Experimental procedures

### Materials

Pyruvate, malate, ethylene glycol tetraacetic acid (Cat# E4378), ADP (Cat# A5285), oligomycin (Cat# 75351), 2,4-dinitrophenol (Cat# D198501), and dibucaine hydrochloride (Cat# D0638) were purchased from Sigma. Tetraphenylphosphonium chloride (Cat# 88060) was from Fluka. Percoll (Cat# 17089101) was purchased from GE Healthcare Bio-Sciences. BSA, free from FFA (Cat# 152401), was purchased from MP Biomedicals. Protease inhibitor cocktail (Cat# 04693124001) was purchased from Roche. Alamethicin (Cat# BML-A150) was from Enzo. All materials were purchased no more than 6 months before use.

### Animals

All procedures with animals were performed in compliance with the US National Institutes of Health Guide for the Care and Use of Laboratory Animals as well as in accordance with the Indiana University School of Medicine Institutional Animal Care and Use Committee approved protocol (# 11385 MD/R). MCU-KO mice were obtained from Dr Toren Finkel (Center for Molecular Medicine, NHLBI, National Institutes of Health) and were maintained on a CD1 (Charles River Laboratories) background, whereas CyD-KO (*Ppif*^*−/−*^, CyD-KO) mice were obtained from Dr Jeffery Molkentin (University of Cincinnati) and maintained on a C57BL/6 (Envigo) background. Breeding colonies were established in the Laboratory Animal Resource Center at Indiana University School of Medicine. MCU/CyD-DKO mice were generated by establishing several parental breeding pairs which consisted of crossing female CyD-KO mice with male MCU-KO mice to yield an F1 generation that was heterozygous for both MCU and CyD. F1 heterozygous mice from different breeders were then crossed with each other to produce an F2 generation that included MCU/CyD-DKO mice. These F2 MCU/CyD-DKO mice were then used to perpetuate the colony and to produce mice that would be used in experiments. The mice were housed under standard conditions in polycarbonate cages, three mice per cage with free access to water and food. For our experiments, 3- to 4-month-old mice were used.

### Genotyping

All offspring were genotyped using a PCR assay on tail DNA to ensure that they were homozygous knockout for both MCU and CyD. Two separate PCR assays were performed on DNA from each mouse to determine the presence or absence of MCU and CyD. The MCU PCR assay was carried out using the following oligonucleotide primers (Invitrogen): forward primer GT F2 (5’ – GGAGTTAAGTCATGAGCTGCTAT – 3’) and reverse primers GT R2 (5’ – CTGGCTTAGTTGGCAGAGTTC – 3’) and V76R (5’ – CCAATAAACCCTCTTGCAGTTGC – 3’) (61) along with Platinum PCR SuperMix (Invitrogen, Cat# 12532024) for amplification. Cycling conditions were initial denaturation at 94 °C for 2 min, followed by 35 cycles (94 °C for 30 s, 60 °C for 30 s, and 72 °C for 30 s), and then 72 °C for 5 min. The WT allele was amplified as a band ∼300 bp, and the MCU-null allele was amplified as a band ∼200 bp. The CyD PCR assay was carried out using the following primers (Invitrogen): Exon3-F (5’ – CTCTTCTGGGCAAGAATTGC – 3’); Neo-F (5’ – GGCTGCTAAAGCGCATGCTCC – 3’); and Exon4-R (5’ – ATTGTGGTTGGTGAAGTCGCC – 3’) along with Platinum PCR SuperMix (Invitrogen) for amplification. The reaction conditions were initial denaturation at 95 °C for 3 min, followed by 35 cycles (95 °C for 30 s, 56 °C for 30 s, and 72 °C for 1 min) and then 72 °C for 10 min. The WT allele was amplified as a band ∼850 bp, and the CyD null allele was amplified as a band ∼600 bp. Reaction products were analyzed on a 1.2% agarose gel run at 100 V for 60 min with Tris acetate–EDTA running buffer containing 1X GelRed nucleic acid gel stain (Biotium, Cat# 41003).

### Isolation of brain nonsynaptic and synaptic mitochondria

Percoll gradient-purified brain nonsynaptic and synaptic mitochondria from CD1, C57BL/6, MCU-KO, CyD-KO, and MCU/CyD-DKO mice were isolated as we previously described (62, 63). Briefly, brains of three mice of each strain were harvested and processed simultaneously. All procedures were performed at 2 to 4 °C. After homogenization of brain tissue in a 15 ml Dounce homogenizer on ice, 30 ml of isolation buffer 1 was added, and diluted homogenate was centrifuged at 2400 rpm for 10 min in a Beckman Avanti J-26XP (Beckman Coulter Life Sciences) centrifuge, rotor JA 25.50 (700*g*). After the first centrifugation, supernatant was centrifuged at 12,500 rpm (18,900*g*) for 10 min. The pellet was resuspended in 35 ml of isolation buffer 2 and centrifuged at 12,200 rpm (18,900*g*) for 10 min. The pellet was then resuspended in 5 ml of isolation buffer 3, and the suspension was layered onto the top of a discontinuous Percoll gradient (26%/40%) contained within Beckman Ultra-Clear centrifuge tubes. The 26% and 40% Percoll solutions were prepared in Percoll Buffer. The suspension, atop the discontinuous Percoll gradient, was then centrifuged at 15,500 rpm (41,100*g*) for 28 min in a Beckman Optima L110K ultracentrifuge, bucket rotor SW41Ti. Following centrifugation, nonsynaptic mitochondria and synaptosomes were each collected separately. To obtain synaptic mitochondria, synaptosomes were subjected to nitrogen cavitation using an ice-cold nitrogen cell disruption vessel (Parr Instrument Co; Cat# 4639) as described previously (62, 63). Briefly, the synaptosomes were transferred to a 10 ml glass beaker on ice and placed into the nitrogen vessel on ice under 1100 psi (7584 kPa) for 13 min. The ruptured synaptosomes were layered onto a discontinuous Percoll gradient (24%/40%) and centrifuged at 15,500 rpm (41,100*g*) for 28 min. Following centrifugation, synaptic mitochondria were collected, and then, both synaptic and previously collected nonsynaptic mitochondria were washed simultaneously. Synaptic and nonsynaptic mitochondria were resuspended in isolation buffer 3 and centrifuged at 15,500 rpm (41,100*g*) for 20 min. Mitochondrial pellets were then resuspended in isolation buffer 3 and centrifuged again at 15,500 rpm (41,100*g*) for 20 min. The nonsynaptic and synaptic mitochondria pellets were then resuspended in isolation buffer 3 and stored on ice. These were stock suspensions of brain nonsynaptic and synaptic mitochondria. The composition of isolation buffer 1: 225 mM mannitol, 75 mM sucrose, 0.1% BSA free from FFA, 10 mM Hepes, pH 7.4 adjusted with KOH, and 1 mM EGTA. BSA was used to preserve mitochondrial integrity (64). The composition of isolation buffer 2: 225 mM mannitol, 75 mM sucrose, 10 mM Hepes, pH 7.4 adjusted with KOH, 0.1 mM EGTA. The composition of isolation buffer 3: 395 mM sucrose, 0.1 mM EGTA, 10 mM Hepes, pH 7.4. The composition of Percoll Buffer: 320 mM sucrose, 1 mM EGTA, 10 mM Hepes, pH 7.4.

### Immunoblotting

Brain nonsynaptic and synaptic mitochondria that were pretreated with Protease Inhibitor Cocktail (Roche) were incubated with NuPAGE LDS sample buffer (Invitrogen, Cat# B0007) plus a reducing agent for 15 min at 70 °C. Bis-Tris gels (4–12%, Invitrogen, Cat# NP0335) were used to separate proteins by electrophoresis (20 μg protein/lane). After electrophoresis, proteins were transferred to a Hybond-ECL nitrocellulose membrane (Amersham Biosciences, Cat# RPN78D). Blots were incubated at room temperature for 1 h in a blocking solution that was composed of either 5% BSA, phosphate-buffered saline, pH 7.2, plus 0.15% Triton X-100 or 5% milk phosphate-buffered saline, pH 7.2, plus 0.15% Triton X-100. After blocking, blots were incubated with either mouse monoclonal anti-CyD antibody (Calbiochem; 1:500; Cat# AP1035), rabbit polyclonal anti-MCU (Atlas Antibodies; 1:1000; Cat# HPA016480), or mouse monoclonal anti-Complex II 70-kDa subunit (Invitrogen; 1:1000; Cat# 459200). Blots were subsequently incubated with either goat anti-mouse or goat anti-rabbit IgG (1:25,000 or 1:20,000, respectively) coupled with horseradish peroxidase (Jackson ImmunoResearch Laboratories) and developed with Supersignal West Pico chemiluminescent reagents (Pierce, Cat# 32106). Molecular mass marker Page Ruler Plus Prestained Protein Ladder (5 μl, Thermo Fisher; Cat# 26619) was used for molecular mass determination of the bands. NIH ImageJ 1.48v software (http://rsb.info.nih.gov//ij) was used for band density quantification.

### Mitochondrial respiration

Mitochondrial respiration was measured under continuous stirring in a 0.4 ml thermostated chamber at 37 °C in the standard incubation medium containing 125 mM KCl, 0.5 mM MgCl_2_, 3 mM KH_2_PO_4_, 10 mM Hepes, pH 7.4, 10 μM EGTA, and 0.1% BSA free from FFA, supplemented with 3 mM pyruvate plus 1 mM malate. In the experiments with Ca^2+^-induced stimulation, BSA, pyruvate, and malate were omitted, and incubation medium was supplemented with 3 mM succinate plus 3 mM glutamate as we described previously (32). The incubation chamber was outfitted with a custom-made Clark-type oxygen electrode and a tightly sealed lid. The slope of the oxygen electrode trace was used to calculate the respiratory rate.

### Mitochondrial Ca^2+^ retention capacity

Mitochondrial Ca^2+^ uptake was measured with a miniature Ca^2+^-selective electrode in a 0.3 ml, continuously stirred chamber at 37 °C. Uptake of Ca^2+^ by mitochondria was indicated by a decrease in Ca^2+^ concentration in the incubation medium. The standard incubation medium contained 125 mM KCl, 0.5 mM MgCl_2_, 3 mM KH_2_PO_4_, 10 mM Hepes, pH 7.4, and 10 μM EGTA and was supplemented with 3 mM pyruvate plus 1 mM malate. In addition, the incubation medium was supplemented with 0.1 mM ADP and 1 μM oligomycin as described previously (65). Ca^2+^ was delivered to mitochondria as 10 μM CaCl_2_ pulses. Data were quantified as Ca^2+^ retention capacity per mg of mitochondrial protein or Ca^2+^ uptake rate per minute per mg of mitochondrial protein.

### Mitochondrial swelling and membrane potential

Mitochondrial swelling was evaluated in a 0.3 ml chamber maintained at 37 °C under continuous stirring by monitoring changes in scattering of 525 nm light in the mitochondrial suspension. The incident light beam was positioned at 180° relative to the photodetector. In our previous study, we showed that Ca^2+^ causes spontaneously reversible mitochondrial swelling in KCl-based incubation medium (66). To avoid this confounding effect, in the present study, the incubation medium used for simultaneous measurements of mitochondrial swelling and membrane potential was based on mannitol-sucrose and contained 215 mM mannitol, 70 mM sucrose, 0.5 mM MgCl_2_, 3 mM KH_2_PO_4_, 10 mM Hepes, pH 7.4, 10 μM EGTA, 3 mM pyruvate, and 1 mM malate. Swelling of mitochondria within the mitochondrial suspension was indicated by a decrease in light scattering. Maximal mitochondrial swelling was induced by alamethicin (30 μg/ml). Alamethicin-induced swelling was considered to be 100% swelling and Ca^2+^-induced swelling was calculated as a percentage of maximal, alamethicin-induced swelling (14). Mitochondrial membrane potential was evaluated using a TPP^+^ sensitive electrode by following TPP^+^ distribution between the incubation medium and mitochondria (67). An increase in TPP^+^ concentration in the incubation medium corresponded to depolarization, whereas a decrease in TPP^+^ concentration in the incubation medium corresponded to polarization.

### Statistics

Data are displayed as mean ± SD of the indicated number of separate experiments. Statistical analysis of the experimental results consisted of unpaired *t* test or one-way analysis of variance with Bonferroni *post hoc* test (GraphPad Prism version 4.0, GraphPad Software Inc). Every experiment was performed using several different preparations of isolated mitochondria.

## Data availability

All data are contained within the manuscript.

## Conflict of interest

The authors declare that they have no conflicts of interest with the contents of this article.
